# Sleep disturbances in craniopharyngioma: a challenging diagnosis

**DOI:** 10.1007/s00415-021-10794-1

**Published:** 2021-09-14

**Authors:** Ramona Cordani, Marco Veneruso, Flavia Napoli, Claudia Milanaccio, Antonio Verrico, Alessandro Consales, Matteo Cataldi, Daniela Fava, Natascia Di Iorgi, Mohamad Maghnie, Maria Margherita Mancardi, Lino Nobili

**Affiliations:** 1 Department of Neurosciences, Rehabilitation, Ophthalmology, Genetics, Maternal and Child Health (DINOGMI), University of Genoa, Genoa, Italy; 2 Department of Paediatrics, IRCCS Giannina Gaslini Institute, Genoa, Italy; 3 Neuro-Oncology Unit, IRCCS Giannina Gaslini Institute, Genoa, Italy; 4 Paediatric Neurosurgery Unit, IRCCS Giannina Gaslini Institute, Genoa, Italy; 5 Child Neuropsychiatry Unit, IRCCS Giannina Gaslini Institute, Genoa, Italy

**Keywords:** Craniopharyngioma, Sleep, Hypothalamic dysfunction, Sleep-related breathing disorders, Hypersomnolence, Narcolepsy, Pitolisant

## Abstract

Craniopharyngiomas are rare solid or mixed solid and cystic tumors that arise from Rathke’s pouch remnants along the pituitary-hypothalamic axis, from the sella turcica to the brain third ventricle. Both the tumor and its treatment can lead to significant neurological and endocrinological complications. Due to the essential role of the hypothalamus in the complex neurophysiologic process of sleep, tumors involving the hypothalamic area may be responsible for disturbances in sleep–wake regulation with alterations in the circadian rhythm, sleep fragmentation, and increased daytime sleepiness. We report two cases of patients with craniopharyngioma, who came to our attention due to the occurrence of episodes characterized by psychomotor slowing and afinalistic limb movements, temporal and spatial disorientation, psychomotor agitation, and oneiric stupor like episodes. A comprehensive clinical data collection and a targeted diagnostic work-up led to a diagnosis of severe sleep disorder characterized by hypersomnia, altered sleep–wake rhythm, and sleep-related breathing disorder. In addition, the polysomnography revealed peculiar alterations in the sleep structure. The diagnostic work-up lead to an accurate differential diagnosis between epileptic seizures and episodes expressions of sleep disturbances. These clinical features can be challenging to diagnose and can lead to misdiagnosis and inappropriate treatment. Diagnosis of sleep disorders is crucial, considering the impact of sleep on general health, cognition, and neuropsychological functioning. These findings support the need to incorporate a comprehensive sleep evaluation in childhood brain tumor involving the suprasellar/hypothalamic region.

## Background

Craniopharyngiomas (CPs) are rare brain tumors of the sellar region that most likely arise from embryonic remnants of the craniopharyngeal duct epithelium, also known as Rathke pouch epithelium [1]. CPs develop along the pituitary-hypothalamic axis, from the sella turcica to the brain third ventricle. Approximately 50% originate in the third ventricle floor, within the infundibulum and/or of tuber cinereum, including the hypothalamus, and spread predominantly into the cavity of the third ventricle [2]. CPs account for 0.5–2.5 new cases per 1 million population per year, with 30% and 50% of all cases presenting during childhood and adolescence [1]. CPs are the most common non-neuroepithelial intracerebral neoplasm in children (< 18 years of age), counting 5%–11% of intracranial tumors in this age group. In childhood and adolescents the adamantinomatous histological type with cyst formation is the most common [2].

Despite a low-grade histological classification (WHO grade I), CP may have a malignant clinical course owing to the hypothalamic-pituitary location and tumor-related and/or treatment-related injury to these areas.

At the time of diagnosis, primary signs are frequently nonspecific manifestations of increased intracranial pressure (such as nausea and headache), visual impairment (losses of visual acuity and visual field) (62%–84%), and endocrine deficits (52%–87%) [2, 3]. Growth impairment have been recognized in patients before diagnosis, while significant weight gain suggesting hypothalamic obesity may occur over time [2].

Treatment for CP may include either radical surgical excision or subtotal resection followed by focal radiation therapy. Overall survival reported in pediatric cohorts ranges from 83% to 96% at 5 years, from 65% to 100% at 10 years, and 62% at 20 years [2]. Despite high survival rates, quality of life is commonly impaired in long-term survivors due mainly to neuroendocrine sequelae caused by the damage of the hypothalamic-pituitary region [1].

The hypothalamus plays a crucial role in regulating vital functions, such as the endocrine system and metabolic processes and in controlling hunger, thirst, and thermoregulation and its injury can lead to significant endocrinological, neurological, and neurocognitive impairments (especially in memory, learning, and school performance areas). Furthermore, the hypothalamus represents a key component of the sleep–wake regulation system being the suprachiasmatic nucleus identified as the master clock of the circadian rhythm [4, 5].

Growing data in the literature demonstrate the increased risk of sleep disorders after the onset of tumors involving the hypothalamic area or after their treatment. Sleep fragmentation, impaired sleep quality and excessive daytime sleepiness (EDS) are commonly reported in CP with a pattern of circadian rhythm characterized by early morning awakening, followed by an extra period of sleep during the afternoon [5–7]. Cases of secondary narcolepsy have also been reported, particularly in children (70% of cases of narcolepsy), with CP being the prevalent type (38%) (8). Moreover, several data prove that suprasellar cancer survivors are more likely to have a greater risk for sleep-disordered breathing [9, 10]. Patients undergoing hypothalamic surgery may lose the ability to downregulate appetite, exhibit abnormal food-seeking behavior and rapid weight gain, and develop obesity, resulting in excessive daytime sleepiness through various mechanisms, such as the increased risk of developing obstructive sleep apnea and augmented circulating levels of pro-inflammatory cytokines which in turn impair sleep [11].

Sleep disturbances assessment plays an essential role in patient management, considering that sleep disturbances can negatively impact several biological functions, cognition, and mood.

Here we describe two cases of patients with CP who developed paroxysmal manifestations, initially misdiagnosed as epileptic episodes, expression of a severe sleep disorder.

## Case reports

### Patient 1

#### Anamnesis

**Fig. 1:**
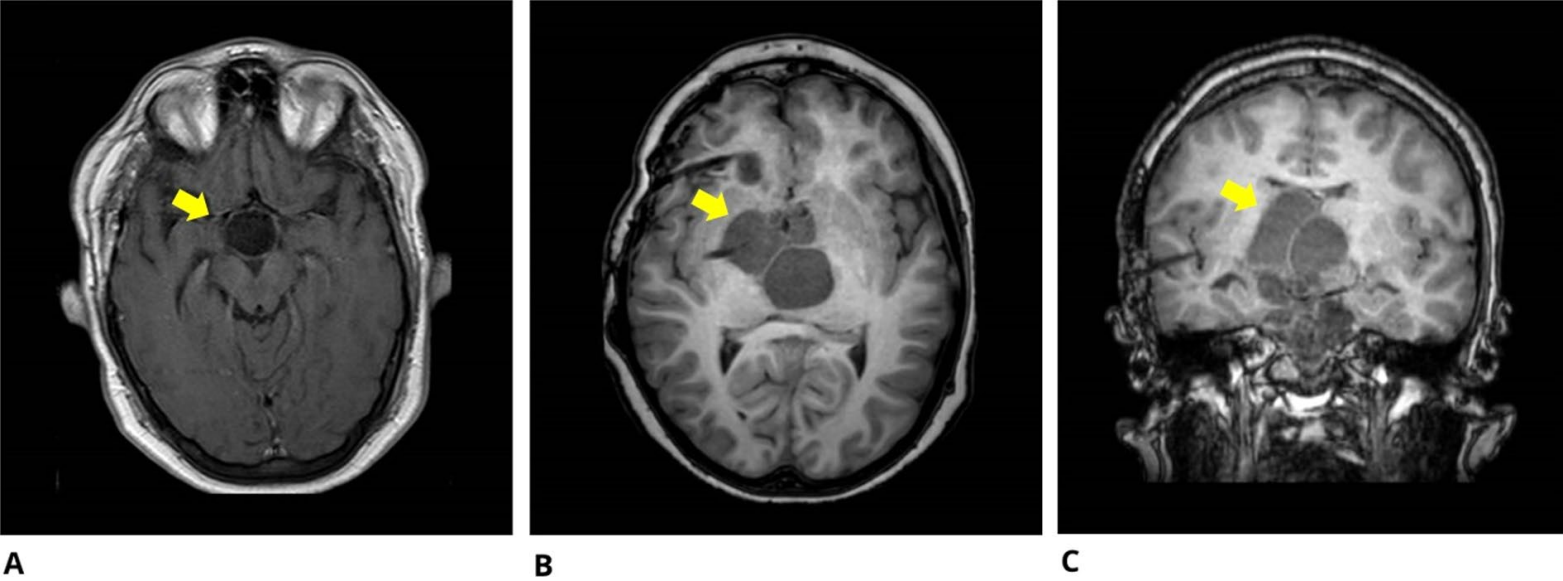
Imaging findings. **a** T1-weighted brain MR imaging shows the result of craniopharyngioma resection surgery in Patient 1 (yellow arrow). **b**–**c** T1-weighted brain MR imaging shows volumetric increase in the post-surgical interpeduncular fossa and right paramedian cysts in Patient 2 (yellow arrow)

The first patient is a previously healthy 19-year-old male diagnosed with CP and hydrocephalus and treated with extensive tumor resection at the age 18 years (Fig. 1a). Subsequently, panhypopituitarism (diabetes insipidus, hypothyroidism, adrenal insufficiency, hypogonadotropic hypogonadism, GH deficiency), necessitating hormone replacement therapy, and hyperphagia and obesity (Body Mass Index, BMI, increased from 25.1 to 41.0 in less than 2 years) occurred. Besides, episodes characterized by psychomotor slowing, sometimes followed by upper limbs afinalistic movements, and episodes of sudden muscle tone loss of the lower limbs appeared and were diagnosed as epileptic seizures in another Neurological Center. However, antiepileptic treatments with phenobarbital, lacosamide, and phenytoin were not followed by a clinical improvement and clinical manifestations worsened over the following months. At first examination in our center, a marked daytime sleepiness with frequent daytime naps, fragmented night sleep, hypnagogic hallucinations and sudden loss of muscle tone while awake were recognized. These lasts occurred in the absence of any evident trigger, such as laughing. Moreover, episodes characterized by bimanual automatic gestures occurring during state of somnolence were reported. In addition, during the medical evaluation, the patient experienced an episode characterized by psychomotor slowing, closing of the eyes, broad movements of the upper limbs, and hardly understandable speech; he seemed to understand the examiner’s questions but did not answer. The clinical evaluation revealed a BMI of 41 and a Mallampati score Class 2.

#### Suspected diagnosis

The presence of symptoms suggestive of sleep disturbances and the occurrence of events that did not resemble epileptic seizures but seemed to be due to sudden falls asleep sometimes associated with hallucinatory phenomena, led to a targeted sleep study.

#### Sleep evaluation and differential diagnosis

**Fig. 2:**
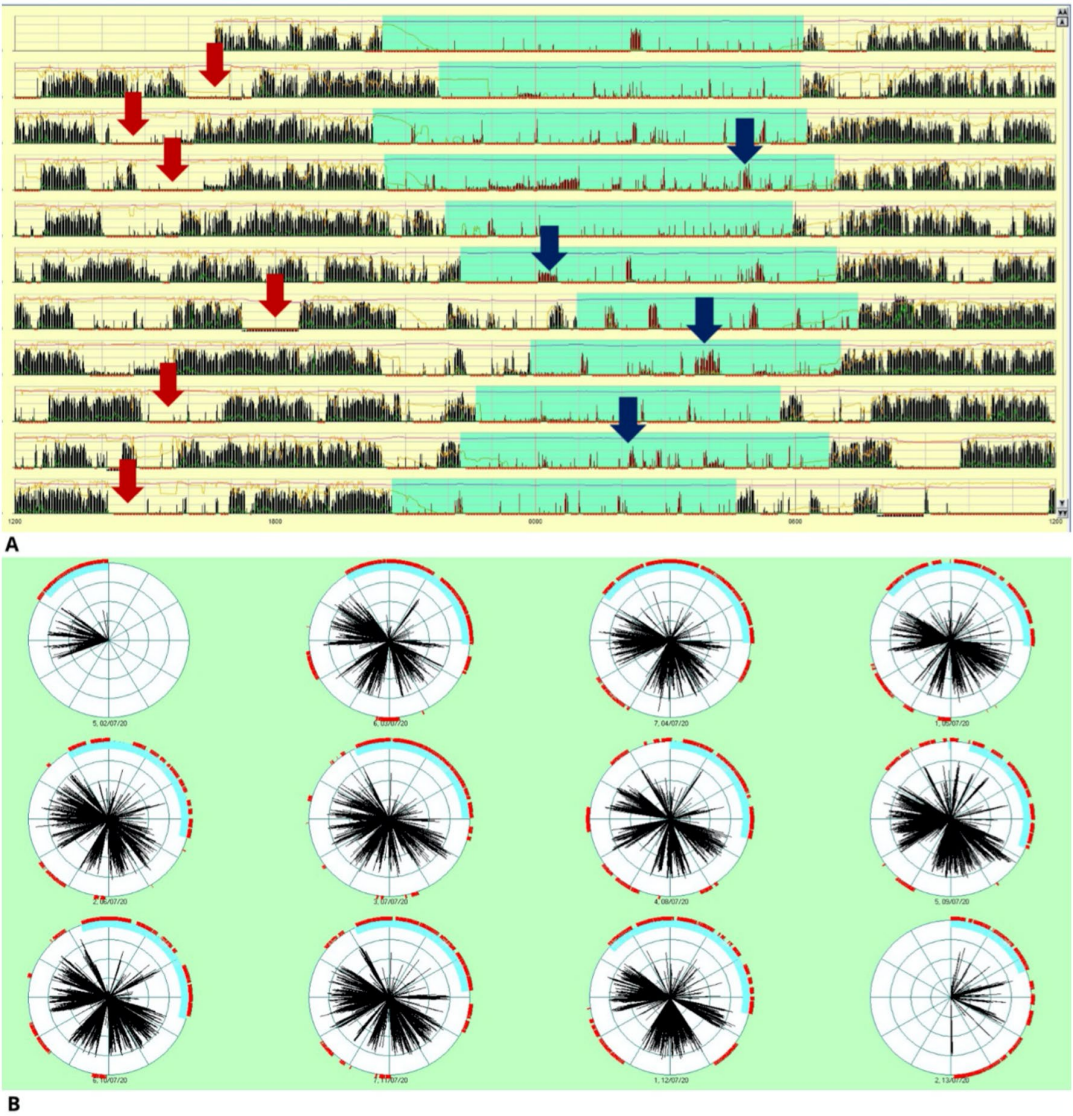
Actigraphy recording: **a** Black bars symbolize movement detected by wrist actigraphy; absence of black bars indicates supposed sleeping periods. Horizontal lines represent consecutive 24-h periods with clock hours indicated on the x-axis. The blue band designates the nocturnal sleep period. Arrows indicate examples of inappropriate presumed daytime sleep (red) or nocturnal arousal (blue). **b** Different graphic representation of the same record. As in the previous figure, black bars symbolize movement detected by wrist actigraphy, and the absence of black bars indicates supposed sleeping periods; the light blue line shows the night hours

**Fig. 3:**
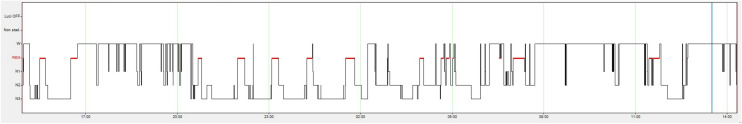
Hypnogram of a 24-h sleep–wake cycle recording, including electroencephalogram, right and left electro-oculogram (EOG), electrocardiogram (ECG) and electromyogram (EMG) of mylohyoideus and left and right tibialis muscles. The PSG recording confirms hypersomnia (13.9 h of total sleep over 24 h), frequent daytime sleep episodes, and night awakenings. Moreover, an altered periodicity of wakefulness and sleep during the 24 h is recognized

The diagnostic work-up included actigraphy recording for 12 days, which demonstrated irregular bedtimes, frequent night-time activity, and inappropriate daytime rest episodes (Fig. 2). The subjective sleepiness assessment using the Epworth Sleepiness Scale (ESS) [12] fit with subjective hypersomnolence (ESS total score = 19, values > 10 are suggestive of excessive daytime sleepiness). Long-term polysomnography (PSG) lasting 24 h confirmed hypersomnia (13.9 h of total sleep over 24 h), frequent daytime sleep episodes, ranging from a few seconds to several minutes (maximum 100 min) with two sleep-onset REM periods (SOREMPs), and prolonged episodes of reduced alertness corresponding to clear-cut sleep onset in the NREM sleep N1 stage (Fig. 3), in the absence of any interictal and ictal epileptic abnormalities. Many of these manifestations were marked in the seizure diary by the mother as corresponding to those reported in the medical history. Therefore, clinical and neurophysiological data allowed to exclude a diagnosis of epilepsy.

Moreover, PSG analysis revealed a severe sleep-related breathing disorder with multiple obstructive apneas, central apneas and hypopneas (Apnea Hypopnea Index -AHI- 50/h; central AHI 40/h, obstructive AHI 10/h) generally associated with desaturation (Oxygen Desaturation Index -ODI- 55/h) mostly occurring during REM sleep. The average SpO2 was 95.6%, time spent with SpO2 < 90% equal to 23.8 min, minimum oxygen saturation value 80%.

Transcutaneous capnography showed a median PCO2 of 42.2 mmHg, time spent with PCO2 > 50 mmHg 0 min, thus excluding nocturnal hypoventilation.

### Treatment and follow-up

A non-invasive overnight ventilatory support (NIV) with Bi-level Positive Airway Pressure (BiPAP) was initiated and a gradual decrease in antiepileptic therapies (first phenobarbital, then phenytoin and lacosamide) was started. At the same time, hormonal replacement therapy was optimized by starting subcutaneous growth hormone and transdermal testosterone therapy, and by switching from twice-daily cortisone acetate to three times daily hydrocortisone.

The patient was reevaluated 4 months later, having ascertained regular daily application of NIV during the previous 2 weeks. The treatment with antiepileptic drugs (AEDs) was gradually reduced but still ongoing. He was assessed by performing polysomnography and subsequent Multiple Sleep Latency Test (MSLT). The polysomnography performed with ventilatory support revealed an improvement in the sleep-related breathing disorder with AHI index reduction (10/h) and a more prominent representation of the NREM sleep N3 phase. Despite the improvement in nighttime sleep, during MSLT, he fell asleep during all tests with an average sleep latency of 4 min and 50 s. One SOREMP occurred. Given the clinical and polysomnographic improvement, the indication for NIV support and the decrease in antiepileptic drugs were renewed. The persistence of the mild sleep-related breathing disorder and the treatment with AEDs could affect the accurate achievement of sleep investigations. Therefore, a re-evaluation of the patient after the AED discontinuation and the regular NIV employment was mandatory.

**Fig. 4:**
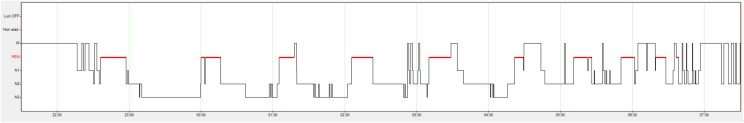
Hypnogram of nocturnal polysomnography. The nocturnal PSG was performed after discontinuation of the antiepileptic drugs and employment of non-invasive ventilation. The sleep onset in REM sleep and a peculiar REM sleep representation characterized by a lack of the physiologic gradual increase in REM episodes duration throughout the night was noticed (27.6% of Total Sleep Time). In addition, many nocturnal awakenings were noted (wake equal to 10.2% of Sleep Period)

During the following months, AEDs were entirely stopped, and NIV was employed routinely. The discontinuation of the AEDs did not lead to a worsening of the paroxysmal episodes. However, subjective daytime sleepiness, albeit subjectively reduced, persisted despite therapeutic adjustments (ESS total score = 16). The PSG performed with ventilatory support revealed a marked improvement in the sleep-related breathing disorder with rare hypopneas, and no apneas (AHI 1.3/h), average SpO2 97%, ODI 2.7/h. Moreover, the nocturnal PSG showed the occurrence of 1 SOREMP and a lack of the physiologic gradual increase in REM episodes duration throughout the night (Fig. 4). In the Multiple Sleep Latency Test (MSLT), the patient fell asleep during all the five tests with an average sleep latency of 6 min and 10 s and 3 SOREMPs occurred. These data allowed a final diagnosis of secondary narcolepsy, and treatment with pitolisant, a histamine 3 receptor inverse agonist, was started. The therapy provided a clinical improvement with reduced daytime sleepiness (ESS total score = 9 after 3 months from the beginning of the treatment).

### Patient 2

#### Anamnesis

The second patient is a boy treated with surgery and radiotherapy for multicystic adamantinomatous CP at the age of 4 years, followed by panhypopituitarism, treated with hydrocortisone, levothyroxine, growth hormone, DDAVP; obesity with hyperphagia (BMI >  + 3SDS since age 5 years, BMI 41 at the last evaluation), and low vision. Over the years, he underwent further surgical treatment to reduce the residual cystic components by placement of several intracystic catheters connected to Ommaya reservoirs allowing liquid aspiration of growing cysts.

At the age of 12 years, during the lockdown period for COVID-19 emergency, he developed episodes characterized by myoclonic jerks, a dreamlike state, temporal and spatial disorientation, and psychomotor agitation. Surmising paroxysmal epileptic episodes, the patient was hospitalized. Patient was referred to Pediatric Neurology for evaluation, and anamnestic data collection revealed a severe loss of environmental and social stimulation during the COVID-19 lockdown period with sleep–wake rhythm dysregulation, fragmented nighttime sleep, marked daytime sleepiness and severe impairment of alertness. Oneiric stupor-like episodes were observed during hospitalization. During these events, the patient performed simple automatic gestures mimicking daily-life activity such as manipulating non-existent objects. When questioned, he appeared confused.

#### Suspected diagnosis

The anamnestic data collection and the observation of the patient's clinical features do not seem to sustain the diagnosis of epilepsy. On the other hand, the severe dysregulation of the sleep–wake rhythm, the marked daytime sleepiness, and the impaired alertness suggested a severe dysfunction of sleep regulation. Therefore, a targeted diagnostic work-up was initiated.

#### Sleep evaluation and differential diagnosis

The diagnostic work-up involved a long-term Video-EEG monitoring including polygraphic measurements, which recorded a severe alteration of the sleep–wake cyclic organization and the sleep structure. The occurrence of short sleep cycles, reduced representation of N2 and N3 NREM sleep phases, frequent episodes of undetermined state of vigilance, and the concurrence of elements typical of different sleep stages (i.e., spindles in a wake-like state and REM sleep, and rapid eye movements quickly followed by N2 NREM sleep) were registered.

From the respiratory point of view, polysomnography revealed a severe breathing disorder with both central and obstructive apneas with desaturations (AHI 19/h, obstructive apneas 2.1/h, central apneas 4.3/h, mixed apneas 0.2/h, hypopneas 12.6/h, ODI 20.5/h, mean oxygen saturation value 94.1%, minimum oxygen saturation value 77%).

We observed episodes characterized by jerks of the trunk and lower limbs and repeated movements of the pelvis occurring in the transition from wake to sleep, with no associated epileptic discharges.

Moreover, frequent nocturnal arousals and parasomnia episodes, accompanied by hypersynchronous delta waves during NREM sleep, could occur both spontaneously or in association with apneas or hypopneas. During some events, the patient sat on the bed in a state of confusion.

Brain MRI showed volumetric increase in the post-surgical interpeduncular fossa and right paramedian cysts (Fig. 1b, c).

Therefore, these investigations confirmed that the patient's complex clinical features were expression of the alteration of the sleep–wake rhythm, and the destruction of the wake-NREM sleep-REM sleep boundaries requiring a multifactorial therapeutic plan.

### Treatment and follow-up

Education on environmental stimulation and proper sleep hygiene was provided. Moreover, an initial pharmacological approach with slow-release melatonin improved night-time sleep but marked daytime sleepiness persisted. Then, the patient underwent surgical treatment of cyst fenestration with sleep–wake rhythm and behavioral improvement. Finally, a non-invasive overnight ventilatory support with bi-level pressure support ventilation was initiated, with a very poor adherence. In the following months, he presented a new worsening of symptoms, and the MRI showed a further volumetric increase in cysts. A few months later the patient died due to complications of acute pancreatitis and pneumonia.

## Discussion

Knowledge about the long-term outcome of tumors involving the hypothalamic area and treatment in pediatric patients has increased in recent years. Hypothalamic dysfunction in children with CP is identified in 35% of patients at diagnosis and in up to 65%–80% of patients after treatment [13]. Long-term complications decrease the quality of life of many long-time survivors [14].

Although growing literature data prove an association between hypothalamic tumors and sleep disturbances, the etiology of sleep disorders is not yet fully understood, and several factors appear to be involved. This report describes two patients with CP who developed “paroxysmal episodes” initially misdiagnosed and subsequently defined as the expression of a severe sleep disorder.

Patient 1 exhibited events characterized by psychomotor slowing with afinalistic movements of the upper limbs initially defined as epileptic seizures. Careful collection of anamnestic data and targeted diagnostic work-up revealed a sleep disorder with hypersomnolence, fragmented nocturnal sleep, hypnagogic hallucinations, sudden loss of muscle tone and a severe sleep-related breathing disorder.

The second patient showed an even more severe phenotype characterized by myoclonic jerks, temporal and spatial disorientation, psychomotor agitation, and oneiric stupor like episodes. A comprehensive clinical data collection allowed to diagnose an altered sleep–wake rhythm, fragmented sleep, reduced alertness, and presumed hallucinatory events. Interestingly, during polysomnography recordings, we documented peculiar findings, such as the destruction of the wake-NREM sleep-REM sleep boundaries and the simultaneous occurrence of elements of different sleep phases. In literature, the asynchronous occurrence of the various components of the different states has been named *status dissociatus* [15], a condition representing a negative prognostic sign, in which elements of one state of being (wake, NREM sleep, and REM sleep) pathologically intrude into another. Extreme dissociation situations that determine the complete loss of any conventionally defined state of being and the circadian pattern determine a condition known as Agrypnia Excitata, a syndrome characterized by loss of sleep and permanent motor and autonomic hyperactivation, related to three different clinical conditions, fatal familial insomnia, Morvan syndrome, and delirium tremens [16].

As for our patients, several conditions may have contributed to the clinical and electrophysiological findings.

Sleep/wake cycles are hypothesized to result from a balance between both circadian and homeostatic influences, with the hypothalamus playing an essential role in regulating the circadian rhythm. Interestingly, current models of sleep regulation suggest that the hypothalamus by hypocretin production plays an essential role in stabilizing wakefulness or sleep after one of these states has been reached through a flip-flop switch [4]. Therefore, hypothalamic injury can cause disturbances in sleep regulation [13]. Literature data reported increased daytime sleepiness in one-third of children with CP, with 40% prevalence in the severely obese children [13]. Irregular bedtimes, frequent nighttime activity, and inappropriate daytime episodes of rest in CP survivors have been recognized by performing actigraphy [17]. A similar irregular pattern was observed by the actigraphy study in Patient 1.

Evidence of both melatonin deficiency and irregular circadian function in CP survivors has been reported by Lipton et al., suggesting a profound dysfunction of the circadian pacemaker of which melatonin rhythm serves as a marker [17]. These authors hypothesized that the complete loss of the circadian melatonin rhythm might indicate a disruption of daytime circadian arousal mechanisms, leaving homeostatic sleep drive unopposed and thus contributing to sleep disruption in these patients. Interestingly, literature data indicate that treatment with melatonin in CP patients with severe daytime sleepiness results in improved daytime sleepiness [18], as confirmed in Patient 2.

Notably, Patient 1 was diagnosed with secondary narcolepsy, and treatment with pitolisant has been started. Cases of secondary narcolepsy in patients with tumors involving the hypothalamic area have been reported in the literature [8]. Interestingly, a low cerebrospinal fluid (CSF) level of hypocretin-1, the wake-promoting neuropeptide typically reduced in patients with narcolepsy type 1, has been detected in a CP patient with symptomatic narcolepsy (sleep latency < 2 min and 3 SOREMPs at the MSLT; no cataplexy). This suggests the possibility that surgical removal of hypothalamic tumor could result in defective production of orexin and consequently induce daytime somnolence [19]. However, in a further study involving five patients who underwent surgical removal of a space-occupying lesion in the hypothalamic/pituitary region, normal CSF hypocretin values were found, thus indicating other factors causing hypersomnia [20]. Interestingly, treatment with wake-promoting agents has shown beneficial effects in CP-related hypersomnolence [10, 21].

Patients with tumors involving the hypothalamic area have an increased incidence of sleep-disordered breathing, both obstructive and central sleep apneas, responsible for excessive daytime sleepiness and reduced alertness [9, 10]. Moreover, apneas and hypopneas can trigger parasomnias, as in the case of Patient 2. Various factors are responsible for the increased incidence of sleep-disordered breathing. First, patients undergoing hypothalamic surgery may develop obesity [22], as both our patients, resulting in an increased risk of sleep-disordered breathing. However, O’Gorman and colleagues who conducted a cross-sectional study of obese CP and obese controls showed that sleep-disordered breathing (including both central and obstructive sleep apneas) was increased in CP patients than in BMI-matched controls [23], underlying the direct role of the hypothalamus in the regulation of respiratory activity [24]. Interestingly, O’Gorman et al. also showed that CP-related respiratory dysfunction is not compensated by hormone replacement therapies, as also observed in our two cases.

In conclusion, on the basis of current knowledge, tumors affecting the hypothalamic area may be responsible for the onset of sleep disorders through various mechanisms. The diagnosis of sleep disorders is crucial, considering that sleep has an essential role on general health, cardiovascular and metabolic well-being, and immune system functioning [25, 26]. Moreover, sleep disturbances are associated with decreased cognitive abilities, increased anxiety and depression, and decreased perceived well-being [27].

The cases detailed in our report highlight that the clinical manifestation of these dysfunctions can be difficult to diagnose and can lead to misdiagnosis and inappropriate treatment that can harm the patient’s health and the quality of life of patients and their families. Furthermore, the diagnosis can be challenging because these patients do not clinically report sleepiness even when it is objectively present [7]. These findings support the need to incorporate comprehensive sleep assessment in survivors from childhood brain tumors involving the suprasellar/hypothalamic region. Furthermore, the occurrence of paroxysmal episodes of uncertain origin requires differential diagnosis between epileptic seizures and events that may be manifestations of sleep disturbances. The accurate anamnestic data collection pointing to examine the clinical features of the episodes and a long-monitoring of sleep–wake activity (long-term Video-EEG, PSG, actigraphy) is crucial.

## References

[1] Daubenbüchel A, Müller H (2015). Neuroendocrine disorders in pediatric craniopharyngioma patients. J Clin Med.

[2] Müller HL, Merchant TE, Warmuth-Metz M, Martinez-Barbera J-P, Puget S (2019). Craniopharyngioma. Nat Rev Dis Primer.

[3] Hoffmann A, Boekhoff S, Gebhardt U, Sterkenburg AS, Daubenbüchel AMM, Eveslage M (2015). History before diagnosis in childhood craniopharyngioma: associations with initial presentation and long-term prognosis. Eur J Endocrinol.

[4] Saper CB, Fuller PM, Pedersen NP, Lu J, Scammell TE (2010). Sleep state switching. Neuron.

[5] Pickering L, Jennum P, Gammeltoft S, Poulsgaard L, Feldt-Rasmussen U, Klose M (2014). Sleep-wake and melatonin pattern in craniopharyngioma patients. Eur J Endocrinol.

[6] Manley PE, McKendrick K, McGillicudy M, Chi SN, Kieran MW, Cohen LE (2012). Sleep dysfunction in long term survivors of craniopharyngioma. J Neurooncol.

[7] Crabtree VM, Klages KL, Sykes A, Wise MS, Lu Z, Indelicato D (2019). Sensitivity and specificity of the modified epworth sleepiness scale in children with craniopharyngioma. J Clin Sleep Med JCSM Off Publ Am Acad Sleep Med.

[8] Weil AG, Muir K, Hukin J, Desautels A, Martel V, Perreault S (2018). Narcolepsy and hypothalamic region tumors: presentation and evolution. Pediatr Neurol.

[9] Mandrell BN, Wise M, Schoumacher RA, Pritchard M, West N, Ness KK (2012). Excessive daytime sleepiness and sleep-disordered breathing disturbances in survivors of childhood central nervous system tumors. Pediatr Blood Cancer.

[10] Crowley RK, Woods C, Fleming M, Rogers B, Behan LA, O’Sullivan EP (2011). Somnolence in adult craniopharyngioma patients is a common, heterogeneous condition that is potentially treatable. Clin Endocrinol (Oxf).

[11] Muscogiuri G, Barrea L, Annunziata G, Di Somma C, Laudisio D, Colao A (2019). Obesity and sleep disturbance: the chicken or the egg?. Crit Rev Food Sci Nutr.

[12] Johns MW (1991). A new method for measuring daytime sleepiness: the Epworth sleepiness scale. Sleep.

[13] Cohen M, Guger S, Hamilton J (2011). Long term sequelae of pediatric craniopharyngioma - literature review and 20 years of experience. Front Endocrinol.

[14] Poretti A, Grotzer MA, Ribi K, Schönle E, Boltshauser E (2004). Outcome of craniopharyngioma in children: long-term complications and quality of life. Dev Med Child Neurol.

[15] Antelmi E, Ferri R, Iranzo A, Arnulf I, Dauvilliers Y, Bhatia KP (2016). From state dissociation to status dissociatus. Sleep Med Rev.

[16] Provini F (2013). Agrypnia excitata. Curr Neurol Neurosci Rep.

[17] Lipton J, Megerian JT, Kothare SV, Cho Y-J, Shanahan T, Chart H (2009). Melatonin deficiency and disrupted circadian rhythms in pediatric survivors of craniopharyngioma. Neurology.

[18] Müller HL, Handwerker G, Gebhardt U, Faldum A, Emser A, Kolb R (2006). Melatonin treatment in obese patients with childhood craniopharyngioma and increased daytime sleepiness. Cancer Causes Control CCC.

[19] Tachibana N, Taniike M, Okinaga T, Ripley B, Mignot E, Nishino S (2005). Hypersomnolence and increased REM sleep with low cerebrospinal fluid hypocretin level in a patient after removal of craniopharyngioma. Sleep Med.

[20] Snow A, Gozal E, Malhotra A, Tiosano D, Perlman R, Vega C (2002). Severe hypersomnolence after pituitary/hypothalamic surgery in adolescents: clinical characteristics and potential mechanisms. Pediatrics.

[21] Marcus CL, Trescher WH, Halbower AC, Lutz J (2002). Secondary narcolepsy in children with brain tumors. Sleep.

[22] Roth CL (2015). Hypothalamic obesity in craniopharyngioma patients: disturbed energy homeostasis related to extent of hypothalamic damage and its implication for obesity intervention. J Clin Med.

[23] O’Gorman CS, Simoneau-Roy J, Pencharz P, MacFarlane J, MacLusky I, Narang I (2010). Sleep-disordered breathing is increased in obese adolescents with craniopharyngioma compared with obese controls. J Clin Endocrinol Metab.

[24] Fukushi I, Yokota S, Okada Y (2019). The role of the hypothalamus in modulation of respiration. Respir Physiol Neurobiol.

[25] Tobaldini E, Fiorelli EM, Solbiati M, Costantino G, Nobili L, Montano N (2019). Short sleep duration and cardiometabolic risk: from pathophysiology to clinical evidence. Nat Rev Cardiol.

[26] Besedovsky L, Lange T, Haack M (2019). The sleep-immune crosstalk in health and disease. Physiol Rev.

[27] Krause AJ, Simon EB, Mander BA, Greer SM, Saletin JM, Goldstein-Piekarski AN (2017). The sleep-deprived human brain. Nat Rev Neurosci.

