# Correction to: Sleep disturbances in craniopharyngioma: a challenging diagnosis

**DOI:** 10.1007/s00415-021-10814-0

**Published:** 2021-09-24

**Authors:** Ramona Cordani, Marco Veneruso, Flavia Napoli, Claudia Milanaccio, Antonio Verrico, Alessandro Consales, Matteo Cataldi, Daniela Fava, Natascia Di Iorgi, Mohamad Maghnie, Maria Margherita Mancardi, Lino Nobili

**Affiliations:** 1 Department of Neurosciences, Rehabilitation, Ophthalmology, Genetics, Maternal and Child Health (DINOGMI), University of Genoa, Genoa, Italy; 2 Department of Paediatrics, IRCCS Giannina Gaslini Institute, Genoa, Italy; 3 Neuro-Oncology Unit, IRCCS Giannina Gaslini Institute, Genoa, Italy; 4 Paediatric Neurosurgery Unit, IRCCS Giannina Gaslini Institute, Genoa, Italy; 5 Child Neuropsychiatry Unit, IRCCS Giannina Gaslini Institute, Genoa, Italy

## Correction to: Journal of Neurology 10.1007/s00415-021-10794-1

The original version of this article unfortunately contained a mistake. The keywords “Narcolepsy” and “Pitolisant” are missing.

Keywords should be 

Craniopharyngioma · Sleep · Hypothalamic dysfunction · Sleep-related breathing disorders · Hypersomnolence · Narcolepsy · Pitolisant

